# A combined screening study for evaluating the potential of exhaled acetone, isoprene, and nitric oxide as biomarkers of lung cancer

**DOI:** 10.1039/d3ra04522f

**Published:** 2023-10-30

**Authors:** Hao Wang, Xin Wei, Yinghua Wu, Bojun Zhang, Qing Chen, Weigui Fu, Meixiu Sun, Hongxiao Li

**Affiliations:** a Institute of Biomedical Engineering, Chinese Academy of Medical Sciences, Peking Union Medical College Tianjin China sunmx@bme.cams.cn lihx@bme.pumc.edu.cn; b State Key Laboratory of Separation Membrane and Membrane Processes, School of Materials Science and Engineering, Tianjin University of Technology Tianjin China; c Department of Cardio-Pulmonary Function, National Clinical Research Center for Cancer, Cancer Institute & Hospital, Tianjin Medical University Tianjin China

## Abstract

*Background*: the early lung cancer (LC) screening strategy significantly reduces LC mortality. According to previous studies, lung cancer can be effectively diagnosed by analyzing the concentration of volatile organic compounds (VOCs) in human exhaled breath and establishing a diagnosis model based on the different VOCs. This method, called breath analysis, has the advantage of being rapid and non-invasive. To develop a non-invasive, portable breath detection instrument based on cavity ring-down spectroscopy (CRDS), we explored the feasibility of establishing a model with acetone, isoprene, and nitric oxide (NO) exhaled through human breath, which can be detected on the CRDS instrument. *Methods*: a total of 511 participants were recruited from the Cancer Institute and Hospital, Tianjin Medical University as the discovery set and randomly split (2 : 1) into training set and internal validation set with stratification. For external validation, 51 participants were recruited from the General Hospital, Tianjin Medical University. Acetone and isoprene from exhaled breath were detected by proton-transfer-reaction time-of-flight mass spectrometry (PTR-TOF-MS), and NO was measured using CRDS. The model was constructed using the ensemble learning method that set eXtreme gradient boosting and logistic regression as the basis model and logistic regression as the senior model. The model was evaluated based on accuracy, sensitivity, and specificity. *Results*: the model achieved an accuracy of 78.8%, sensitivity of 81.0%, specificity of 70.0%, and area under the receiver operating curve (ROC, AUC) of 0.8341 (95% CI from 0.8055 to 0.8852) in the internal validation set. Furthermore, it attained an accuracy of 66.7%, sensitivity of 68.2%, specificity of 65.5%, and AUC of 0.6834 (95% CI from 0.5259 to 0.7956) in the external validation set. *Conclusion*: the model, established with acetone, isoprene, and NO as predictors, possesses the ability to identify LC patients from healthy control (HC) participants. The CRDS instrument, which simultaneously detects acetone, isoprene, and NO, is expected to be a non-invasive, rapid, portable, and accurate device for early screening of LC.

## Abstract 2

NO and VOCs are combined to establish a diagnostic model. The feasibility of modelling acetone, isoprene, and NO is validated, which provides a basis model for the development of a small CRDS lung cancer diagnostic instrument.

## Graphical abstract

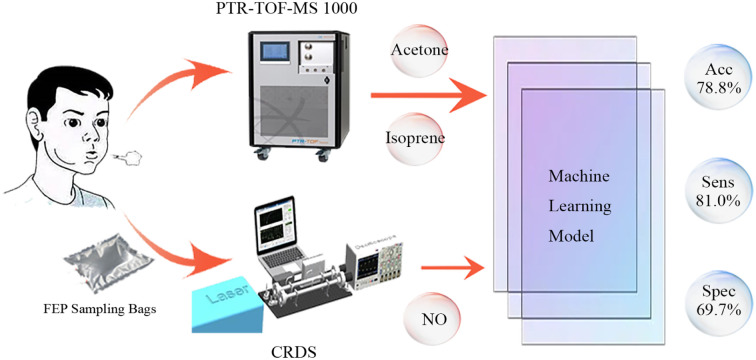

## Introduction

Lung cancer (LC) has a high incidence and mortality rate among other cancers.^1^ The leading cause of its high mortality is that most LC cases are at an advanced stage at the initial diagnosis.^2^ According to clinical statistics, the five year survival rate of stage IV non-small cell lung carcinoma (NSCLC) was only 5%^2^ compared with 60% for stage I NSCLC patients who underwent a surgery.^3^ Therefore, early screening plays a crucial role in reducing lung cancer mortality. Currently, computed tomography (CT), which has contributed 20% relative risk reduction of LC death, is widely applied in the clinics.^4^ However, CT has limitations of a high false positive rate, over-reliance on the experience of the radiologist, radiation, and a complex screening process^5–7^. In this study, we discuss the feasibility of establishing a low-complexity, portable, rapid, and non-invasive early screening strategy from the perspective of exhaled breath biomarkers.

Breath analysis (BA) based on volatile organic compounds (VOCs) from exhaled breath is a potential screening strategy that has the advantages of being non-invasive, rapid, and accurate.^8^ Of all the VOCs, acetone and isoprene have been reported as more than five times more effective biomarkers of LC diagnosis in many studies.^9–12^ Mitrayana *et al.* investigated the exhaled acetone, ethylene, and ammonia as biomarkers of LC using photoacoustic spectroscopy and found a significant difference in acetone (*p*-value < 0.01) between LC patients and healthy control (HC) groups.^13^ Tsou *et al.* analyzed 116 VOCs, including acetone, in breath samples and developed a model that attained a sensitivity of 96% and specificity of 88%.^14^ Wang *et al.* demonstrated 16 LC-related VOCs, including isoprene, and established a diagnosis model with a sensitivity of 89.2% and specificity of 89.1%.^15^ In addition, nitric oxide (NO), an inorganic constituent, has been demonstrated as related to LC.^16^ Liu *et al.* reported that eNO in LC patients has a significantly higher level than that in the healthy control (HC) subjects (*p*-value < 0.01).^17^

The common breath analysis method is mass spectrometry (MS), laser spectroscopy, and nanosensors. Because MS (such as gas chromatography-MS (GC-MS),^18^ proton transfer reaction-MS (PTR-MS),^19^ PTR-time of flight-MS (PTR-TOF-MS),^20^ membrane inlet mass spectrometry (MIMS),^21^ and solid phase microextraction GC-MS (SPME-GC-MS)^22^) have the advantages of simultaneously detecting a wide range of VOCs,^23^ they are widely used for VOC selection during the predictive model development. In terms of instrument volume, mass spectrometry instruments are generally difficult to use as portable instruments due to the need to operate in a vacuum environment, which makes them larger in size (such as secondary electrospray ionization MS (SEI-MS)^24^ and PTR-TOF-MS). The inlet mass spectrometry (IMS) method, called atmospheric pressure mass spectrometry, can effectively reduce the size of the instrument but still leaves much to be desired in terms of measurement accuracy. In terms of measurement accuracy, mass spectrometry measurements are usually semi-quantitative, but there are still instruments (such as SEI-MS and MIMS) that achieve more accurate measurements through pre-processing and other methods. In this study, we used PTR-TOF-MS as the detection instrument to measure the concentrations of acetone and isoprene. Laser spectroscopy and nanosensors are being used in the development of diagnostic equipment due to their ability to quantitatively measure the concentration of specific VOCs as well as their advantages of smaller size and ease of operation. In addition, they can extend the range of biomarkers measured to the inorganic species. In this study, we used cavity ring-down spectroscopy (CRDS), a laser spectroscopy technique, to measure the NO concentration in our model.

Currently, the measurement of acetone, isoprene, and NO with the limit of detection (LoD) of 57 ppb,^25^ 0.47 ppb,^26^ and 7.4 ppb,^27^ respectively, was achieved using CRDS in our group. Meanwhile, our group also investigated the predictive value of individual molecules of NO and isoprene for LC with an accuracy of 72.8%^28^ and 77.9%^29^ respectively, using CRDS. Based on our previous research, acetone, isoprene, and NO were selected to explore the feasibility of diagnosing LC patients by establishing a model in this study.

## Methods

### Subjects

**Fig. 1:**
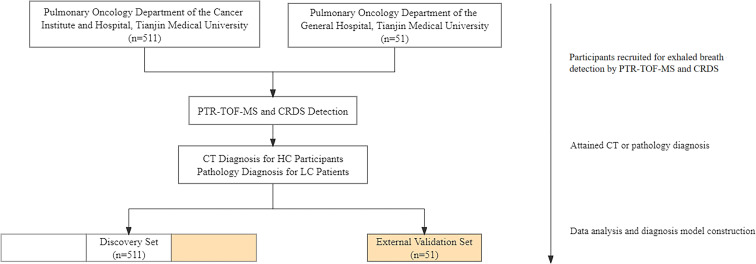
Participant recruitment, exhaled breath analysis, and data analysis process.

In this study, 510 participants were recruited (411 LC patients and 99 HC participants) as a discovery set with the method of randomized controlled trial (RCT) in the Pulmonary Oncology Department of the Cancer Institute and Hospital, Tianjin Medical University, during the period from February 2019 to January 2020. 51 adults, including 22 LC patients and 29 HC participants were recruited as an external validation set with the method of RCT in the Pulmonary Oncology Department of the General Hospital, Tianjin Medical University from October 2020 to June 2021. The recruitment process is shown in Fig. 1. LC patients were confirmed by pathology, and HC participants were examined by computed tomography (CT). The exclusion criteria were as follows: (i) participants under 18 years of age. (ii) Participants who have a tumor history. (iii) Participants who have received chemotherapy (with anti-cancer drugs), immunotherapy, hormone therapy, or radiation therapy. (iv) Women who are already pregnant. (v) Participants with diabetes. Demographic, pathological, and stage characteristics are shown in Table 1.

**Table 1:** Characteristics of the participants

Characteristics	Discovery set		External validation set	
	Lung cancer (*n* = 411)	Healthy control (*n* = 99)	Lung cancer (*n* = 22)	Healthy control (*n* = 29)
Age	59.6 ± 8.5	43.4 ± 13.1	60.8 ± 9.7	29.0 ± 9.0
BMI	24.3 ± 3.3	24.1 ± 3.2	23.1 ± 2.6	22.8 ± 3.5
Male (%)	235 (57.2%)	63 (63.6%)	15 (68.2%)	9 (31.0%)
Fasting (%)	153 (37.2%)	91 (91.1%)	26 (100.0%)	28 (96.6%)

**Smoking (%)**				
Smokers	63 (15.3%)	28 (28.3%)	0 (0%)	3 (10.3%)
Ex-smokers	168 (40.9%)	7 (7.1%)	8 (36.4%)	0 (0%)
Non-smokers	180 (43.8%)	64 (64.6%)	14 (63.6%)	26 (89.7%)

**Category (%)**				
Adenocarcinoma	262 (63.7%)	NA	21 (95.5%)	NA
Squamous cell carcinoma	57 (13.9%)	NA	0 (0%)	NA
Small-cell lung cancer	40 (9.7%)	NA	0 (0%)	NA
Missing	52 (12.7%)	NA	1 (4.5%)	NA

**Stage (%)**				
Carcinoma *in situ*	17 (4.1%)	NA	0 (0%)	NA
I	137 (33.3%)	NA	18 (81.8%)	NA
II	74 (18.0%)	NA	2 (9.1%)	NA
III	67 (16.3%)	NA	2 (9.1%)	NA
IV	75 (18.2%)	NA	0 (0%)	NA
Missing	41 (10.0%)	NA	0 (0%)	NA

### Breath collection

Exhaled breath collection was divided into two parts: direct collection and sampling bag collection, which served PTR-TOF-MS and CRDS detection. All subjects were required to breathe deeply before sampling and breathe out three times spaced 5–6 seconds in the buffered end-tidal (BET) online sampling of the PTR-TOF-MS instrument, which presented sufficient time interval to stabilize the waveform. Subsequently, the exhaled breath was also collected into a fluorinated ethylene propylene (FEP) bag and detected with the CRDS instrument in the laboratory. Sampling bags were repeatedly rinsed four times with high-purity nitrogen gas prior to each collection. The volume of the exhaled breath gas did not exceed 80% of the capacity of the sampling bag for each collection. From collection to laboratory detection, the storage time of the collected gases did not exceed three hours.^27^ The specific collection flow is shown in Fig. 1.

### PTR-TOF-MS and CRDS detection

The exhaled breath samples were measured by PTR-TOF-MS,^30^ which provided acetone and isoprene concentrations, and CRDS,^28^ which provided NO concentrations. The PTR-TOF-MS (PTR-TOF-MS 1000, Ionicon Analytik GmbH, Innsbruck, Austria) in combination offers a real-time online quantitative analysis of the end-tidal fraction of exhaled breath gas with an ultra-low detection limit (LoD < 10 pptv) and high resolution (>2000*m*/Δ*m*). It achieved accurate and absolute quantified detection of VOC concentration with IONICON-exclusive genuine PTR-MS soft ionization technology by proton transfer from H_3_O^+^ and a precise control of the ion source and drift tube parameters. Since different stains are required to measure different substances, this study only measured the concentration of NO by CRDS to prevent the concentration changes over time. The CRDS instrument constructed by our group attained a detection limit of 7.4 ppb, and the baseline stability of the system was 0.52% with good repeatability, stability, and real-time performance. The specific structure, principle, and performance of the CRDS instrument were reported in our previous work.^31^

### Model construction

Machine learning is an appropriate option for classification tasks with simple and quantifiable features. With the aim of adequately exploring the relationship between acetone, isoprene, nitric oxide, and lung cancer, an integrated learning approach was selected to construct the model. The model construction is shown in Fig. 3. The eXtreme gradient boosting (XGBoost) with the “gbtree” core and linear regression (LR) algorithms were opted as the basis model and the LR algorithm was used for the senior model selected.

**Fig. 2:**
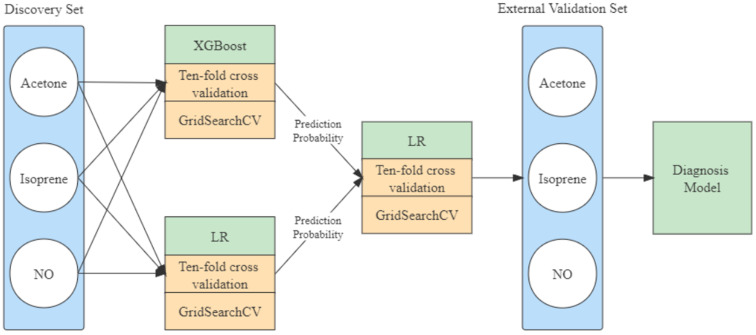
Diagnosis ensemble model construction. The ensemble model sets the LR and XGBoost algorithm as the basis model's baseline and the XGBoost algorithm as the senior model's baseline. The prediction results of the basis model are used as a feature in the senior model (LR: linear regression, XGBoost: eXtreme gradient boosting, GridSearchCV: grid hyper-parameters search algorithm).

The LR parameter settings of penalty and maximum iterations and the XGBoost parameter settings of estimators, maximum depth, and gamma values are critical for training. The grid hyperparameters search was selected to obtain the best parameter combination in the pre-given range of parameters. The min–max and square root normalization were applied to the discovery set as a preprocessing strategy before training. The class weight was switched to the balanced mode, and balanced accuracy with ten-fold cross-validation was chosen as the criteria. The construction of the ensemble model is shown in Fig. 2.

### Statistical analysis

Accuracy, sensitivity, and specificity were obtained to evaluate the diagnostic model. The receiver operating curve (ROC) and area under the ROC (AUC) were also calculated to evaluate the diagnostic model's performance. Feature importance was chosen to evaluate the diagnosis ability for acetone, isoprene, and NO. The correlation matrix of the participants' demographic information, acetone, isoprene, and NO is shown with the heat map.

### Ethics approval

The study was conducted according to the guidelines of the Declaration of Helsinki and was approved by the Ethics Committees of the Cancer Institute and Hospital, Tianjin Medical University, and Tianjin Medical University General Hospital. The present trial was registered with the Institutional Review Board of the Chinese Clinical Trial Registry (registration number: chiCTR1900023659), and all methods were conducted in accordance with relevant guidelines and regulations. Informed consent was obtained from all participants.

## Results

### Characteristics of the participants

A total of 510 participants were recruited in the discovery set from the Pulmonary Oncology Department of the Cancer Institute and Hospital, Tianjin Medical University, including 411 LC patients and 99 HC participants. In the discovery set, 298 were men, and the mean (SD) age was 56.4 (11.5) years. In the external validation set, 51 participants were recruited from the Pulmonary Oncology Department of the General Hospital, Tianjin Medical University, including 22 LC patients and 29 HC participants. In the external validation set, 24 were men, and the mean (SD) age was 42.7 (18.0) years.

### PTR-TOF-MS and CRDS detection

**Fig. 3:**
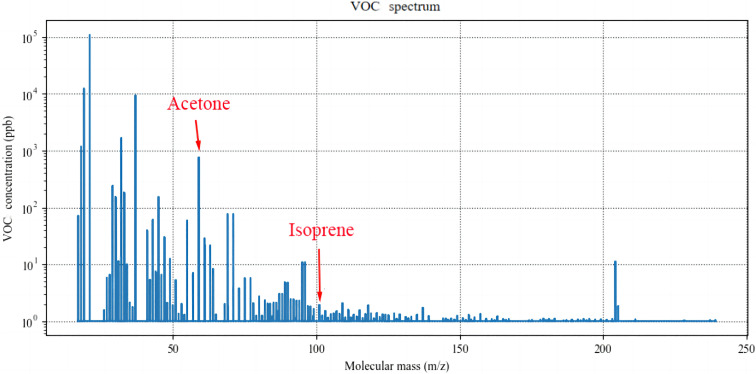
The mass spectrum of the participant reflects the concentration of the VOCs. The molecular mass of acetone is 59.0491 and that of isoprene is 69.0699. (VOCs: volatile organic compounds).

**Fig. 4:**
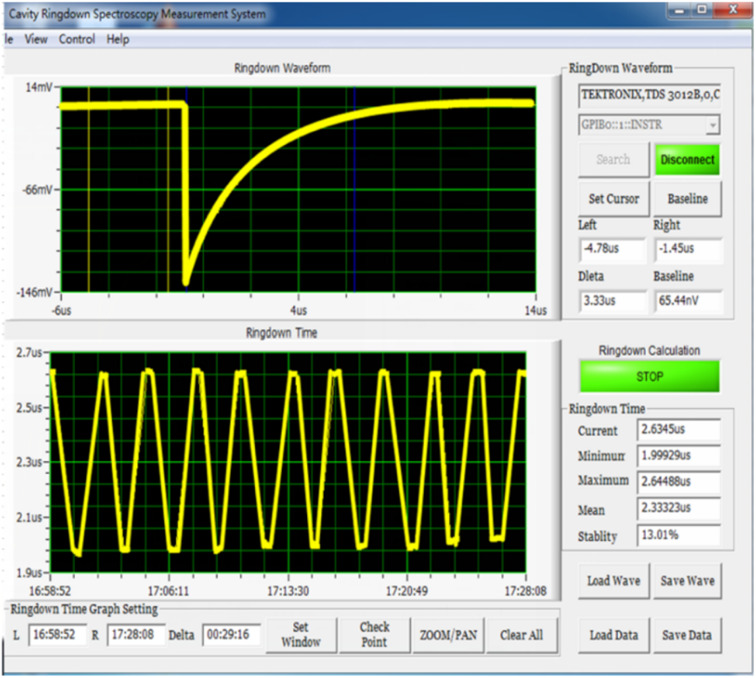
NO detection spectrum of the participants using CRDS. The concentration of NO was calculated using the ring-down waveform and ring-down time. (CRDS: cavity ring-down spectroscopy).

For each participant, the detection was performed three times and the calculated stable waveform average value as the concentrations of acetone, isoprene, and NO were calculated. The detection spectra are shown in Fig. 3 and 4.

### Evaluation models in the discovery set and the external validation set

**Fig. 5:**
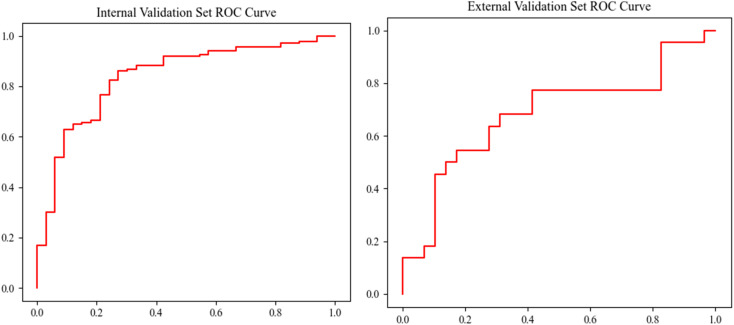
The ROC of the internal and external validation sets.

**Fig. 6:**
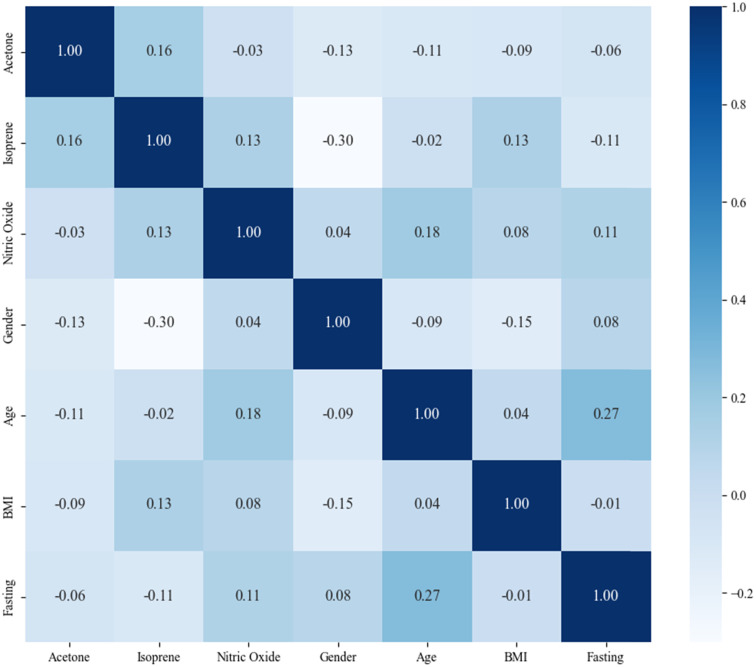
The heat map of the correlation matrix between participants' demographic information and features. Positive values represent a positive correlation and negative values represent a negative correlation. The closer the value is to 1, the higher is the correlation between the two features.

The discovery set was split into a training set and the internal validation set (2 : 1) with stratification. The internal validation attained an accuracy of 78.8%, a sensitivity of 81.0%, a specificity of 69.7%, and an AUC of 0.8341 (95% CI from 0.8055 to 0.8852). The external validation with the model trained on the whole discovery set attained an accuracy of 66.7%, a sensitivity of 68.2%, a specificity of 65.5%, and an AUC of 0.6834 (95% CI from 0.5775 to 0.7758). The validation performances of the model are shown in Table 2, and the ROC is shown in Fig. 5. The correlation matrix of the participants' demographic information, acetone, isoprene, and NO is shown in Fig. 6. The results in the heat map indicate that acetone, isoprene, and NO were not significantly correlated with the participants' demographic information.

**Table 2:** Performance of the model in the internal and external validation sets

Criteria	Internal validation (95% CI)	External validation (95% CI)
AUC	0.8341 (from 0.8055 to 0.8852)	0.6834 (from 0.5259 to 0.7956)
Accuracy	0.7882 (from 0.7529 to 0.8124)	0.6667 (from 0.5578 to 0.7755)
Sensitivity	0.8102 (from 0.7479 to 0.8273)	0.6818 (from 0.537 to 0.7962)
Specificity	0.6970 (from 0.6536 to 0.8448)	0.6552 (from 0.5399 to 0.8129)

### Feature importance

**Fig. 7:**
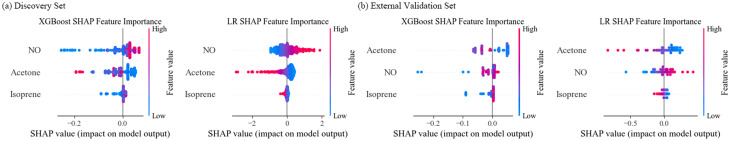
Important features in the (a) discovery set and (b) external validation of SHAP. The consistency of the colors of the points on the same side of the vertical axis represents the feature's ability to discriminate between the data sets.

Feature importance reflects the contribution of acetone, isoprene, and NO. The Shapley addictive explanations (SHAP) method, used to evaluate the ensemble model, indicates the classification capabilities of each feature by giving each participant a prediction probability. The feature importance of the basic model, which directly reflects the relationship between features and outcome variables is shown in Fig. 7. The important features in the discovery set are NO, acetone, and isoprene are shown in Fig. 7(a). The important rankings of the features in the external validation set are acetone, NO, and isoprene, as shown in Fig. 7(b). According to the SHAP, LR possesses better diagnostic ability than XGBoost.

## Discussion

BA has the advantages of being non-invasive and rapid, which is comfortingly accepted. Most BA studies have established a diagnostic model with over 10 VOCs based on the MS detection technique.^15,32–34^ Despite MS supporting multiple VOC detection and more VOCs leading to a more accurate diagnosis model, the expensive cost, complex operation, and large size of MS prevent the universality of the BA diagnosis model. In this study, based on our previous studies of CRDS measurements of acetone, isoprene, and NO, we explored the feasibility of models with low feature numbers and illustrated the potential of acetone, isoprene, and NO to diagnose LC. Meanwhile, CRDS, which can detect inorganic constituents enlarges the biomarkers range of LC.

The results show that the model established using acetone, isoprene, and NO can diagnose LC patients effectively. A portable, accurate, real-time LC diagnosis instrument based on CRDS is feasible, and CRDS provides the potential to establish a more accurate diagnosis model by supporting inorganic constituent detection.

The model established by acetone, isoprene, and NO attains an accuracy of 78.8%, sensitivity of 81.0%, and specificity of 69.7% in the internal validation, and an accuracy of 66.7%, sensitivity of 68.2%, specificity of 65.5% in the external validation. This indicates that the model possesses an acceptable ability to identify LC patients and HC participants according to the internal validation results and is proven to be promotable in other centers according to the external validation results. Cai *et al.* used the electronic nose-GC method that attained an accuracy of 82.8%, sensitivity of 76.0%, and specificity of 94.0% in the internal validation set with 23 VOCs.^33^ Phillips *et al.* used GC-MS and attained an accuracy of 84%, sensitivity of 75.4%, and specificity of 85% in the internal validation set with 8 VOCs.^18^ Compared with other studies, our model performed similarly to those of other studies and required fewer features. Meanwhile, our previous studies on the separate detection of acetone, isoprene, and nitric oxide using CRDS made it possible to measure them simultaneously with the CRDS. The CRDS has the potential to be miniaturized into a screening device that can be widely used in the community and at home. The heat map of the correlation matrix, as shown in Fig. 6, indicates that acetone, isoprene, and NO are independent of each other and not significantly correlated with participants' demographic information.

According to the SHAP analysis, the XGBoost model provides a good recognition for HC patients and the LR model identifies LC patients with a high confidence. The ensemble model integrates the advantages of the XGBoost and LR models and provides a better performance. As shown in Fig. 7, acetone and nitric oxide exhibited significant sorting ability, which contributed to the high difference in prediction probability between LC and HC.

The statistical analysis demonstrated that the model established using acetone, isoprene, and NO is a potential and feasible method to apply in the CRDS instrument as a diagnostic model. Limited by the problem that the current CRDS is incapable of measuring three substances simultaneously, this study chose to model acetone and isoprene detected by mass spectrometry and NO detected by CRDS. In the next step, we will integrate more measurable effective substances as LC biomarkers into the CRDS instrument and resolve the difficulty in measuring multiple substances simultaneously.

The limitations of this study will be validated in the next research stage. First, the sensitivity and specificity need to be improved. Second, the participant demographics in the discovery and external validation sets need to be balanced to further evaluate the model's performance across different subgroups. The model will be validated and calibrated on a larger and multi-center set in the next research stage.

## Conclusion

In this study, we initially validated the feasibility of a lung cancer screening model using acetone, isoprene, and NO. Based on our preliminary work, we have laid the foundation for the subsequent development of a portable and accurate CRDS LC screening instrument.

## Conflicts of interest

There are no conflicts to declare.

## Supplementary Material
